# American Medical Association, Section on Stomatology

**Published:** 1902-10

**Authors:** 


### AMERICAN MEDICAL ASSOCIATION, SECTION ON STOMATOLOGY.

(Continued from page 684.)

*Dr. R. R. Andrews.*—Had I known, when requested to write something about the embryology of the dental pulp, that Dr. Latham was preparing a paper upon the histology of the pulp, I doubt if I should have attempted to write. She can cover that subject so well. The embryology only goes a short distance towards perfect development. My idea was to give a description of the normal dental pulp.

(For Dr. Andrews's paper, see page 621.)

The following Nominating Committee was appointed: Dr. J. L. Williams, Boston; Dr. G. V. I. Brown, Milwaukee; Dr. F. A. Bogue, New York.

Discussion of the papers of Drs. Andrews and Latham was postponed to the morning session of June 11.

Adjourned to June 11, ten A.M.

*Second Session, Wednesday, June 11, 1902.*

The meeting was called to order by the chairman.

A paper entitled "Some Notes concerning Preparation of Teeth for Microscopic Study," was read by Dr. Martha Anderson, of Moline, Ill.

(For Dr. Anderson's paper, see page 588.)

DISCUSSION.

*Dr. Vida A. Latham.*—I would like to say that this work is going on with the work of Dr. Talbot to discover some ways of preparing the teeth that will give results superior to those already quoted. Those quoted are from general histologic work, and are inferior. A tooth that is decalcified has to lose some structure. If we lose the dental structure, we also lose the pulp structure. The nerve-fibres we do not fully understand. We have to be very cautious in making specimens; one batch will stain beautifully, the next not at all. Two pulp-stones passing through the same solution will have results entirely opposite. Even hæmotoxylon will not stain the same in two sections of pulp. We see so many pathological changes that we have been astonished with the different results. We have not yet obtained a normal standard of pulp area. Our results bear out one or two of the results given in Professor Gysi's paper. We consider the importance of this structure equal to that of enamel and dentine. The peculiarity of the pulp lies in its blood supply. The term myxomatous tissue is in dispute. If we use that term we are out of the bounds of histology, because it is not normal tissue. The term analogue is complex. The term dentinal papilla is unsuitable.

*Dr. Eugene S. Talbot.*—I do not think it is possible to study the diseases of the teeth without first studying the evolution of the pulp. There is no structure in the human body that has undergone such a change as the dental pulp, and yet there is nothing taught in our dental schools regarding the matter, so far as I know. The dental pulp has reached its highest development in man before it commences to develop the dentine. From that time on the pulp recedes, and there is no question in my mind from the studies I have made that it becomes a degenerate organ from that period. I think that is the reason, as Dr. Latham said, we are unable to ascertain what a normal pulp is. I question, indeed, whether we have such a thing as a normal healthy pulp, and that, I believe, is the reason we are unable to get two slides or two specimens alike of the same pulp. I do not believe there is any structure in the human body like it in variety of histologic conditions.

*Dr. William Knight.*—It has occurred to me, in regard to injections into the capillaries, that the siphon method with a very dilute saline mixture would be very satisfactory. The vessels after being cleaned out become very lax, and afterwards such injections as are desired can be made. In that way we can get very fine capillary injections. I make this as a suggestion, and not as the result of practical work in histology. It works very beautifully in injecting the vessels of the mesentery.

*Dr. M. L. Rhein.*—The difficulty in securing a proper microscopic specimen of the pulp is one that confronts us continually, and I do not think the solution of the scientific treatment of the pulp will be solved except by microscopic preparation. Clinical experience has taught us empirically the solution of many mooted points, but there is much to be cleared up by pulp specimens under the microscope. I do not know of any one more competent to cope with the solution of this problem than Dr. Latham with all the ingenuity and skill she has given to the work.

*Dr. H. H. Butterfield.*—Coming in as the paper was nearly finished, I heard a statement regarding the pulp-stain, and I would like to ask the conclusion.

*Dr. Latham* (answering Dr. Butterfield's inquiry about the staining of the pulp-stones, reading from her paper.).—Dr. Anderson says the stones are stained dark brown as the nerves. If they are pulp-stones, why do they stain? We have used the teeth the moment they are extracted, and after they have been extracted for some time and are dry and mummified, and again, while still in the jaw. We have tried to collect some thousands of teeth to prepare by every possible method. Later we hope to tabulate these results. My paper yesterday was not a conclusion, but a statement of what others have said on the subject, showing the differences of opinion. This question on pulp-stones in Dr. Anderson's paper was brought out because I have said to her, "How is it you get the staining in the pulp-stones?" She said she did not know. I asked her to take these pulps and break them down, and she used almost every acid known, but without success. Professor Black says he can break them down. Some do not break down. If they do not break by any chemicals, what is the structure? One peculiarity in my photographs is that the nerve-fibres work something like this (diagram on board).

Another point is that the pulp-stones are more common in the apex. The trunk of a nerve-fibre coming down and the pulp being split makes a spiral formation around the pulp-stone. I have other specimens again where the nerve-fibres run up outside, following the fibre and avoiding the pulp-stone. Why do the nerve-tissues take the stain the same as the pulp-stones? One argument is that they are of nerve formation. This is as far as I have gone.

*Dr. M. L. Rhein.*—Do not the formative nerve-cell fibres take the stain?

*Dr. Latham* (in answer to Dr. Rhein's inquiry regarding the staining of formative nerve-cell fibres).—If I remember rightly, Dr. Andrews says formative fibres. He evades the question just as we all have done. They do not stain alike. Then, again, the matrix which is formed does not stain the same as the odontoblastic fibres. These are points about which I am almost in despair. If these are not normal teeth, then we do not want to work with them. If they are normal teeth, we want them. The matrix, as I understand, is possibly the connective-tissue material underneath the layer of odontoblasts. Why these pulp-stones take the stain of the normal tissue is a big question. Dr. Paul makes the statement that the odontoblast is not a formative cell. Gentlemen like Dr. Andrews, who have made the study of the dentine a specialty, are better able than I, who am studying the formative pulp, to answer these questions. The origin of the odontoblastic cell I believe the key-note to the matter. I would be greatly obliged if any of you gentlemen can tell me who quoted the odontoblastic cell as a mesoblastic cell. Where that statement originated I do not know. We know that when a paper is first written it becomes altered by other journals publishing it. Each man who reads the paper draws his own conclusion; that is, he establishes his own theory. I would naturally make another paper fit with mine. It is a fault, but then it is done.

It seems to me that if we agree that the odontoblast is secretive and is a dentine-forming organ, we put it in the hypoblastic layer, because on analogy the glands of the stomach are first secretive. In the lower animals it is on the same basis. Then, if it secretes, it should belong to a secretive organ. We know that all the glands are hypoblastic. The date of the development of the odontoblastic layer I do not know.

*Dr. R. R. Andrews.*—The first formation of the dentine takes place in a mass of connective tissue when there is apparently nothing but the embryonic cells. There is a sort of gelatinous substance thrown out, and these same cells that were connective cells differentiate. They must be different from the connective cells because they were right in the midst of embryonic tissue. The only question is about the basic membrane.

*Dr. Latham.*—A new paper I have now seems to point out that they are nerve-tissue. If they are the terminal nerve-endings they cannot be connective tissue. The results of Morgenstern have not been reproduced by other investigators. In my research I hope by the improved technic to get the fibre into the cell, and in that way prove the point in dispute.

The Nominating Committee reported as follows: Dr. M. L. Rhein, New York, Chairman; Dr. Eugene S. Talbot, Secretary.

At this point the discussion of the papers of Drs. Andrews and Latham, postponed from the day before, was taken up.

(To be continued.)

